# Physician Perception and Diagnosis of Intestinal Parasitic Infections among Patients with Gastrointestinal Symptoms in Ghana

**DOI:** 10.1155/2021/6695313

**Published:** 2021-04-28

**Authors:** Verner N. Orish, Saviour Anorkplim Simpiney, Sylvester Yao Lokpo, Percival D. Agordoh, Duniesky Martinez Lopez, Thelma M. Alalbila, Adekunle Sanyaolu

**Affiliations:** ^1^Department of Microbiology and Immunology, School of Medicine, University of Health and Allied Sciences, Ho, Volta Region, Ghana; ^2^Department of Physician Assistant, School of Medicine, University of Health and Allied Sciences, Ho, Volta Region, Ghana; ^3^Department of Medical Laboratory Sciences, School of Allied Health Sciences, University of Health and Allied Sciences, Ho, Volta Region, Ghana; ^4^Department of Nutrition and Dietetics, School of Allied Health Sciences, University of Health and Allied Sciences, Ho, Volta Region, Ghana; ^5^Department of Internal Medicine, School of Medicine, University of Health and Allied Sciences, Ho, Volta Region, Ghana; ^6^Pharmacy Practice Department, School of Pharmacy, University of Health and Allied Sciences, Ho, Volta Region, Ghana; ^7^Federal Ministry of Health, Abuja, Nigeria

## Abstract

This study evaluated physicians' perception and diagnosis of intestinal parasitic infections (IPI) in patients with gastrointestinal (GI) symptoms. This cross-sectional survey used a Google form questionnaire distributed online. Demographic and clinical practice information was solicited, including if “IPI was considered as a diagnosis in the last patient seen,” “if stool investigation was requested among the last patients seen,” and physicians' perception of the burden of IPI in the country. Using Pearson chi-square and multivariate logistic regression analysis, we tested the significance of the associations of the job cadre of the physicians and their perception of the IPI burden with consideration of IPI as a diagnosis in the last patient seen, request for stool investigation in the last patient seen, and overall frequency of the request for stool investigation. Ultimately, 184 physicians responded. The majority agreed to “often seeing patients with GI symptoms” (156, 84.7%), “not considering IPI among the last patient seen” (106, 57.6%), and “not requesting stool investigation among the last patient seen with symptoms” (136, 73.9%). House officers (81, 44.2%) constituted the highest proportion of physicians who considered IPI as a diagnosis among the last patient seen (39, 48.1%, *p* = 0.05). Most physicians (138, 75%) considered IPI as a burden in Ghana. They constituted significant proportions of the physicians who considered IPI as a diagnosis among their last patients seen (65, 83.3%, *p* = 0.02) and were twice more likely to consider IPI as a diagnosis among the last patients seen than their colleagues who did not consider IPI as a burden in Ghana (AOR 2.26, *p* = 0.04). The consideration of IPI as a diagnosis among patients with GI symptoms and request for stool investigations was low among physicians in this study. Further engagements with physicians in Ghana are needed to help improve their diagnosis of IPI in patients with GI symptoms.

## 1. Introduction

Intestinal parasitic infections (IPI) are common parasitic infections reported globally but predominantly seen in tropical areas especially in places bedeviled by poor socioeconomic situations like poor sanitation, inadequate potable water supply, open defecation, and poor personal hygiene [1–3]. There are about 3 billion people infected globally, with endemic infections seen in many African countries where one-third of the population is infected with IPI especially among school-age children and preschool children [3, 4]. IPI seldom lead to death among those infected but have been linked with worrisome morbidities, contributing significantly to the burden of the poor physical and mental development in children and over 35 million disability-adjusted life years (DALYs) globally [1, 5, 6].

The majority of IPI often have asymptomatic presentations [3]. However, a significant proportion of infections usually manifest with some symptoms, like vomiting, diarrhoea, and abdominal pains [3, 7]. Asymptomatic or infections with mild symptoms usually do not seek care in healthcare facilities [8]. Those who do, with moderate and severe disease, present with symptoms indistinguishable from other gastrointestinal disorders caused by other pathogens [8, 9]. For physicians to adequately manage cases of IPI that come to the hospital with symptoms, a high index of suspicion is needed to make an accurate diagnosis [10]. To do this, it is necessary for the physician to at least consider IPI as a diagnosis in these patients and follow up with a request for stool investigation because using clinical symptoms alone might not be appropriate [10]. However, IPI in patients with gastrointestinal (GI) symptoms like vomiting, diarrhoea, and abdominal pain is not often immediately considered by the physician. Thus, empirical treatment is given with an antibiotic without recourse to stool routine investigations [11, 12]. This practice of not considering IPI among patients with GI symptoms and subsequently not requesting stool investigation among physicians can obscure the true burden and morbidity of IPI in the populations [12–14].

In Ghana, IPI is a well-recognized public health problem [15–17]. This burden attracts a vibrant policy response from the Ministry of Health involving an active periodic deworming program among school children [18]. This periodic deworming exercise in Ghana might be yielding the desired result as evident by the low prevalence of IPI reported in some studies [17, 19–21]. However, to effectively ascertain the burden of IPI and the impact of periodic deworming, it is important to constantly investigate the presence of intestinal parasites among persons who come to the hospital with gastrointestinal symptoms. This will be hugely dependent upon physicians who attend to these patients vis-à-vis if they consider IPI as a cause of their patient's symptoms and if they request diagnostic stool investigation. No study in Ghana has looked at the diagnostic practice of the physicians concerning IPI; hence, this study intends to evaluate physicians' perception and how they diagnose cases of IPI in patients with gastrointestinal symptoms.

## 2. Materials and Methods

### 2.1. Study Design

This study was a cross-sectional study involving the electronic distribution of questionnaires to physicians across the country aimed at finding out their perception and diagnostic approach of IPI.

### 2.2. Study Site

This study took place in Ghana, a sub-Saharan African country in West Africa. It is located between the latitude of 7.9465°N and a longitude of 1.0232°W, bordered by Francophone countries with Togo on the East, Ivory Coast on the West, and Burkina Faso on the North as well as the Gulf of Guinea and the Atlantic Ocean on the South. Ghana, as of August 2020, has an estimated population of about 31 million people with about 49% of these people living in rural communities and about 38% living in urban slums, with many using public toilets and practicing open defecation [22, 23].

Ghana has about three thousand doctors working in over 800 government-sponsored facilities. Over half of these facilities serve as primary care health facilities with about 61 being district hospitals and others being tertiary hospitals [24].

### 2.3. Study Population and Data Collection

This study targeted registered physicians of all cadres and specialty working in all the 16 regions of the country. The contacts including email or phone numbers of registered and practicing physicians were obtained from various official social media platforms, and Google form questionnaires were sent to about 1500 doctors, between January and March 2018. The Google form questionnaires clearly and briefly explain the purpose of the study with an appeal to voluntarily answer the questions provided.

Some of the questions asked were brief demographics, the number of years in practice and location of practice whether urban or rural, job cadre and area of specialization, frequency of encounter with patients with GI symptoms, consideration of IPI as a diagnosis in the last patient seen with GI symptoms, request of stool microscopy in the last patient seen with GI symptoms, and overall frequency of request for stool investigation.

### 2.4. Sample Size Calculation

A purposive sampling method was employed in this study to recruit some physicians from all 16 regions in active practice in both the private and public sectors. The Cochran formula was used to calculate the sample size using *P* as 50% which is an estimated prevalence of physicians who consider IPI as a diagnosis in the last patient seen (since actual prevalence is unknown), *Z* value of 1.96 with a confidence interval of 95%, and allowable error of 0.05.

(1) $$\begin{array}{c} n=\frac{Z^{2}\left(P\right)\left(1-P\right)}{{\left(AE\right)}^{2}}=385. \end{array}$$

A modified Cochran formula was then used, imputing *n* = 385 as the Cochran calculated sample size with population of 3000 doctors (*N*).

(2) $$\begin{array}{c} n=\frac{n}{1+\left(n/N\right)}=\frac{385}{1+\left(385/3000\right)}=341. \end{array}$$

This resulted in a minimum sample size of 341.

### 2.5. Data Analysis

Data obtained from the Google forms were exported to the Statistical Package for Social Sciences (SPPS) version 25 software where the analysis was carried out. Frequency distribution was done for all variables, and a chi-square analysis was done to test the significance of the association of the job cadre of the physicians and their perception of the burden of IPI with the consideration of IPI as a diagnosis in the last patient seen with GI symptoms, request of stool investigations in the last patient seen, and the overall frequency of stool request investigations. Multivariate logistic regression analysis was done to further analyze the association between physician perception of the burden of IPI and consideration of IPI as a diagnosis in the last patient seen with symptoms. All statistical analyses were conducted using a 95% confidence interval with a significance set at *p* ≤ 0.05.

## 3. Results

### 3.1. Demographic Characteristics of Physicians

A total of 184 physicians responded to the Google form questionnaire sent to them. Of these, 128 were males (69.6%) and 56 were females (30.4%) (Table 1). The majority of the doctors were between the ages of 20 and 40 (138, 91.4%), had no clinical specialty (155, 84.2%), and work in urban settings (146, 79.3%). There were 81 house officers (57 house officers and 24 senior house officers) and 89 medical officers (53 medical officers, 26 senior medical officers, and 10 principal medical officers).

### 3.2. Characteristics of Clinical Practice of Physicians

Table 2 shows the practice characteristics of the physicians. The majority of the physicians often encounter patients with gastrointestinal symptoms (156, 84.8%), with most seeing an average of 1-4 patients in a week (75, 40.8%). The most common symptoms encountered were a combination of vomiting, diarrhoea, and abdominal pain (98, 53.3%). Eighty-seven of the physicians considered children (<5, 47, 25.5%; >5, 40, 21.7%) with symptoms as likely suffering from IPI. With respect to the last patient seen, the majority of the physicians did not consider IPI as a possible diagnosis (106, 57.6%) and the majority did not request stool investigation (136, 73.9%). Generally, most of the physicians sometimes requested stool investigations (88, 47.8%) when they see patients with GI symptoms, while a few always requested stool investigation to confirm their diagnosis of IPI (26, 14.1%) in patients with GI symptoms. The majority of the physicians used albendazole or mebendazole to treat IPI (175, 95.1%). The majority also used ciprofloxacin in combination with metronidazole to empirically treat patients (120, 65.2%). Most of the physicians considered IPI a burden in Ghana (138, 75%).

**Figure 1:**
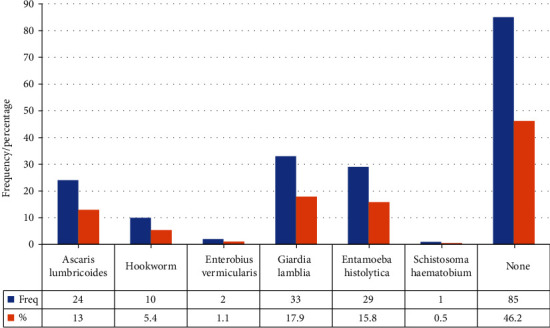
Intestinal parasites seen by physicians.

Most physicians who sometimes request stool investigations in patients with GI symptoms reported that they had no laboratory confirmation of intestinal parasites in stool specimens investigated (85, 46.2%), while intestinal protozoa were the most commonly seen parasites among the physicians who had laboratory confirmation of intestinal parasites in stool specimens investigated (*Giardia lamblia*, 33, 17.9%; *Entamoeba histolytica*, 29, 15.8%) (Figure 1).

### 3.3. General Characteristics of Physicians Stratified by Job Cadre

Table 3 shows the characteristics of physicians stratified by the job cadre. There was a significant association between the job cadre of physicians and the tendency to consider IPI as a diagnosis in the last patient seen with GI symptoms. House officers significantly considered IPI as a diagnosis in the last patient seen with GI symptoms (39, 48.1%) more than medical officers (38, 42.7%) or specialists (1, 7.1%) (*p* = 0.05). No specialist in this study saw a patient with GI symptoms within the past 7 days, and only 7.1% of them always requested stool investigation in patients with GI symptoms they encountered compared to the other cadre of physicians; however, these findings were not significant (*p* = 0.8, *p* = 0.4).

### 3.4. Association between the Perception of IPI as a Burden and the Tendency among Physicians to Consider IPI as a Diagnosis in the Last Patient Seen

Table 4 shows the association between physicians who considered IPI as a burden and those who considered IPI as a diagnosis. Of the physicians who considered IPI as a diagnosis in their last patient seen, the majority significantly agreed that IPI is a burden in Ghana (65, 83.3%, *p* = 0.021). Physicians who considered IPI as a burden were 2 times more likely to consider IPI as diagnosis among the last patient seen than their colleagues who did not consider IPI as a burden (adjusted odds ratio 2.26, 95% CI 1.043-4.910, *p* = 0.021).

### 3.5. Relationship between Duration of Practice with Consideration of IPI as a Diagnosis, Confirmation with Stool Investigation, and Frequency of Stool Request among Physicians

**Figure 2:**
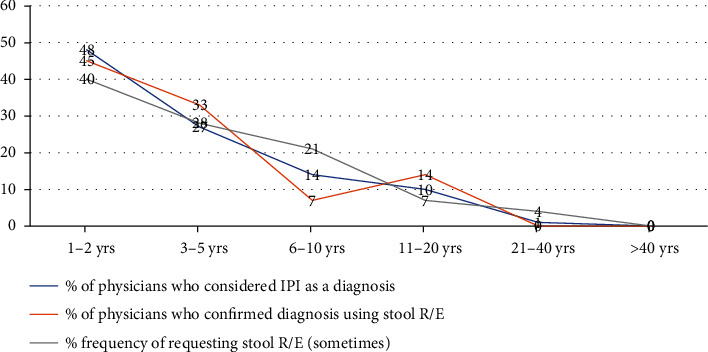
Relationship of the duration of years of practice distribution with consideration of IPI as a diagnosis, confirmation with stool R/E, and frequency of request of stool investigation.

Figure 2 shows an inverse relationship between the duration of practice with consideration of IPI as a diagnosis, confirmation of IPI using stool investigation, and frequency of stool investigation request among physicians. A higher proportion of physicians who had fewer years of practice considered IPI as a diagnosis in the last patient seen, requested stool investigation to confirm the diagnosis in the last patient seen, and frequently requested stool investigation compared to physicians with more years of practice.

## 4. Discussion

Patients with GI symptoms were commonly encountered by the physicians in this study. About 84% of the physicians surveyed claimed they see these patients often and only about 11% claimed not to have seen any of such patients within the past 7 days. Studies have shown that gastrointestinal symptoms are indeed one of the commonest reasons for hospital visits and hospitalizations among patients in both developed and developing countries [25, 26]. Diarrhoea no doubt is the most dramatic of gastrointestinal symptoms because of the potential to cause mortality especially among children [27]. In this study, the majority of the physicians highlighted diarrhoea as the most common symptom seen; however, this was in combination with vomiting and abdominal pain. The combination of vomiting, diarrhoea, and abdominal pain is a very common presentation in patients with gastroenteritis, a common gastrointestinal illness caused by bacteria, viruses, and parasites [28]. These symptoms offer very little in helping the physician arrive at a diagnosis of IPI as these symptoms are incapable of distinguishing the pathogens involved in the gastrointestinal illness [8, 9]. However, the chronicity, recurrence, or persistence of symptoms as well as if the symptoms are seen in children and the immunocompromised may be suggestive of IPI [11]. The majority of the physicians in this study considered children and HIV-infected people as categories of patients whose symptoms can be caused by IPI. In Ghana, most studies have reported the vulnerability of children and the HIV-infected people to IPI [15, 16, 21, 29, 30].

Only about 42% and 26% of physicians considered IPI as a diagnosis and requested stool investigation among the last patient seen with GI symptoms, respectively, and only 14% of physicians claimed to always request stool investigations in patients with GI symptoms. Though no study in Ghana has evaluated the clinical practices of diagnosis of IPI among physicians, findings from this study highlight the possibility that physicians might not be adequately considering and requesting stool investigations to confirm the diagnosis of IPI among patients presenting with GI symptoms. In areas endemic for intestinal parasites, there is a significant contribution of IPI in patients with GI symptoms [31, 32]; therefore, it is apropos for physicians to consider IPI in patients with GI symptoms and always request a stool investigation to confirm the diagnosis. With this study showing several antibiotics prescribed by the physicians, it is possible that many physicians might be overprescribing antibiotics in the empirical treatment of patients with GI symptoms which might be contributing to the nagging problem of antibiotic resistance [10, 33–35].

This study shows physicians of lower job cadre like house officers and medical officers as well as physicians with lower years of experience constituted higher proportion of physicians who considered IPI as a diagnosis among the last patient seen with GI symptoms, requested stool investigation to confirm IPI in the last patient seen, and more frequently utilized stool investigations to confirm IPI in patients with symptoms. The lower job cadre and lower years of experience of house officers and medical officers in this study might have influenced them to consider simple and common causes of diseases and to rely on stool investigations to help confirm their diagnosis, compared to the specialists and physicians with more years who might rely on years of personal experience [36].

This study showed that a significant proportion of the few physicians who considered IPI as a diagnosis among the last patients they saw with GI symptoms were physicians who perceived IPI as burden in the country. Physicians who considered IPI as a burden were 2 times more likely to consider IPI as a diagnosis in patients with GI symptoms. Diagnosis of a condition in a patient has been found to be influenced by the physician's knowledge of local conditions [36]. IPI is endemic in Ghana with studies reporting cases in both urban and rural settings in the country [15, 16, 21], and this was firmly supported by 75% of the physicians in this study who agreed that IPI is a burden.

This study showed that intestinal protozoa were the most common parasites reported to have been seen by the physicians from stool investigation, with *Giardia lamblia* (18%) mostly seen followed by *Entamoeba histolytica* (16%). *G lamblia* and *E histolytica* are common intestinal protozoa reported in some asymptomatic children and children with diarrhoea in Ghana [15, 17, 37–40]. Helminths like *Ascaris lumbricoides* and hookworm have also been reported in studies in Ghana [19, 20]. However, the majority of the physicians did report not seeing any parasite in stool investigations (46%). This finding might be linked to the sensitivity issues of the wet mount stool microscopy technique which is the most prevalent stool investigation used in many health facilities in IPI endemic areas including Ghana [9, 10, 12, 21].

This study has some few limitations that are worth mentioning. This study strictly depended on the information and responses furnished by the physicians and these might be blighted by recall bias and responses that can only be verified by direct observation of physician clinical practices. Secondly, the relatively small sample size might make it difficult to generalize the findings of this study. However, despite these limitations, the findings from this study highlight very important issues that are noteworthy and need further investigations.

## 5. Conclusions

Despite majority of the physicians encountering patients with GI symptoms and agreeing that IPI is a burden in Ghana, only few of these physicians considered IPI in the patients seen and fewer still employed stool investigations to help confirm their diagnosis. Further studies are needed to understand the situation, and possible engagement with physicians might be needed to help improve the practice of diagnosis of IPI in patients presenting with gastrointestinal symptoms.

## Figures and Tables

**Table 1:** Demographic and general characteristics of the physicians.

Characteristics	Frequency	%
Age range (years)		
20-40	138	91.4
40-60	9	6
>60	4	2.6
Gender		
Male	128	69.6
Female	56	30.4
Duration of practice (years)		
1-2	72	43.4
3-5	48	28.9
6-10	28	16.9
11-20	12	7.2
21-40	5	3
>40	1	0.6
Cadre		
House officer	57	31
Senior house officer	24	13.1
Medical officer	53	28.8
Senior medical officer	26	14.1
Principal medical officer	10	5.4
Consultant	14	7.6
Specialty		
No specialty	155	84.2
Surgeon	5	2.7
Physician	11	6
Obstetrician and gynaecologist	8	4.3
Paediatrician	3	1.6
Dentist	2	1.1
Location of practice		
Urban	146	79.3
Rural	38	20.7

Data are represented as numbers (%) of physicians.

**Table 2:** Characteristics of clinical practice of physicians.

Characteristics	Frequency	%
Frequency of encounter of patients with GI symptoms		
Often	156	84.8
Rarely	27	14.7
Not at all	1	0.5
No. of patients seen in the past 7 days		
>7	55	29.9
5-7	34	18.5
1-4	75	40.7
None	20	10.9
Common symptoms patients presented		
Vomiting (V)	3	1.6
Diarrhoea (D)	23	12.5
Constipation (C)	8	4.3
Anorexia (A)	6	3.2
Nausea (N)	4	2.2
Abdominal pain (AP)	19	10.3
D & A	4	2.2
D & AP	5	2.7
V & D	14	7.6
V, D & AP	98	53.3
Category of patients with symptoms increasing suspicion of intestinal parasitic infections		
Children < 5	47	25.5
Children > 5	40	21.7
Pregnant women	5	2.7
Elderly	3	1.6
HIV	6	3.2
Child+HIV	36	19.7
Child+pregnant+HIV	28	15.2
Elderly+pregnant+child	11	6
Was IPI considered in the last patient with GI symptoms?		
Yes	78	42.4
No	106	57.6
Confirm diagnosis using stool investigation in the last patient seen		
Yes	48	26.1
No	136	73.9
Frequency of stool R/E request		
Always	26	14.1
Often	16	8.7
Most times	10	5.4
Sometimes	88	47.8
Rarely	40	21.7
Not at all	4	2.2
Criteria for giving anthelminthic		
Symptoms	89	48.3
Stool R/E	23	12.5
Common parasite in locality	15	8.2
Symptoms+stool R/E	57	40
Drug of choice for intestinal parasite		
Albendazole/mebendazole	175	95.1
Metronidazole	4	2.2
Praziquantel	1	0.5
None	4	2.2
Antibiotics of choice for gastrointestinal symptoms		
Ciprofloxacin	18	9.8
Metronidazole	33	17.9
Amoxicillin	2	1.1
Cefuroxime	3	1.6
Ceftriaxone	2	1.1
Tetracycline	1	0.5
Doxycycline	1	0.5
Cipro+metronidazole	120	65.2
Is intestinal parasite a burden in the country?		
Yes	138	75
No	46	25

Data are represented as numbers (%) of physicians.

**Table 3:** Characteristics of physicians stratified by cadre.

Characteristics	House officers (81) (%)	Medical officers (89) (%)	Specialist (14) (%)	*p* value
Intestinal parasitic infection considered in last patient GI symptoms				
Yes	39 (48.1)	38 (42.7)	1 (7.1)	
No	42 (51.9)	51 (57.3)	13 (92.9)	0.05
Confirm diagnosis using stool investigation				
Yes	22 (27.2)	26 (29.2)	0 (0)	
No	59 (72.8)	63 (70.8)	14 (100)	0.1
Frequency of stool R/E request				
Always	12 (14.8)	13 (14.6)	1 (7.1)	
Often	8 (9.9)	8 (8.9)	0 (0)	
Most times	3 (3.7)	7 (7.7)	0 (0)	0.41
Sometimes	38 (46.9)	43 (48.3)	7 (50)	
Rarely	18 (22.2)	17 (19.1)	5 (35.7)	
Not at all	2 (2.5)	1 (1.1)	1 (7.1)	
Any parasite seen from the result of stool R/E				
Some	41 (50.6)	58 (65.2)	9 (64.3)	
None	40 (49.4)	31 (34.8)	5 (35.7)	0.16
Frequency of encountering patients with GI symptoms				
Very often	27 (33.3)	25 (28.1)	2 (14.3)	
Often	44 (54.3)	50 (56.2)	9 (64.3)	0.8
Rarely	11 (13.5)	13 (14.6)	3 (21.4)	
Not at all	0 (0)	1 (1.1)	0 (0)	

Data represented as number (%) of physicians; *p* value significant at ≤0.05. GI = gastrointestinal; R/E = routine examination.

**Table 4:** Association between the perception of IPI as a burden and considering IPI as a diagnosis in the last patient with gastrointestinal symptoms.

Characteristics		Is IPI a burden in Ghana?		*p* value	Adjusted OR^∗^ (95% CI)	*p* value
		Yes (%)	No (%)			
Was IPI considered in the last patient with GI symptoms?	Yes	65 (83.3)	13 (16.7)	0.021	2.263 (1.043-4.910)	0.04
	No	73 (68.9)	33 (31.1)			

^∗^Adjusted for job cadre, duration of practice, and specialty.

## Data Availability

The datasets used during the current study are available from the corresponding author on request.

## Abbreviations

AOR: Adjusted odds ratio
CI: Confidence interval
GI: Gastrointestinal
IPI: Intestinal parasitic infections
R/E: Routine examination
SPSS: Statistical Package for Social Sciences.
